# An accurate method of determining a single-plane osteotomy to correct a combined rotational and angular deformity

**DOI:** 10.1007/s11751-015-0222-6

**Published:** 2015-04-09

**Authors:** James Youngman, Dimitri Raptis, Khalid Al-Dadah, Fergal Monsell

**Affiliations:** Department of Trauma and Orthopaedic Surgery, University College London Hospital, 235 Euston Road, London, NW1 2BU UK; University Hospital Zurich, Zurich, Switzerland; Imperial College London, London, UK; Bristol Royal Hospital for Children, Bristol, UK

**Keywords:** Osteotomy, Correction, Deformity, Realignment, Single cut

## Abstract

Conventional osteotomy used for the correction of deformity is performed out of the plane of deformity creating a wedge either opening or closing when the deformity is corrected. Deformity that is a combination of rotation and angulation exists in a single plane that is oblique to the coronal, sagittal and axial planes depending on the magnitude of deformity measured in each plane. Accurate planning and a simple method of finding this oblique plane operatively is presented. This method starts by finding the bisector of angulation. This is marked by a wire that lies in the plane of angulation and along the bisector of angulation. The saw blade is rotated about this bisector axis according to the proportion of angulation and rotation. There is no second reorientation of the saw blade required making the final plane much easier to define. This single-plane oblique osteotomy allows accurate realignment of the limb.

## Introduction

Bone deformity is a combination of angulation and rotation commonly. Coronal plane angular deformity (varus and valgus) is seen on AP radiographs, and sagittal plane angular deformity (procurvatum and recurvatum) is seen on lateral radiographs. Deformity measured on both AP and lateral radiographs can be resolved into a single oblique plane. Imaging at right angles to this oblique plane of deformity shows the maximum deformity (as seen in profile), whereas imaging into the plane of deformity shows no deformity.

The plane of a pure angular deformity lies in the long axis of the bone. Rotational deformity is angulation in the axial plane. Rotational deformity is not seen on coronal or sagittal plane imaging directly and can only be measured directly on axial imaging. The plane of rotational deformity is transverse to the long axis. Osteotomy in this plane is used to correct pure rotational deformity allows full bone contact after correction.

A transverse or axial plane osteotomy used for correction of angulation creates an opening wedge requiring bone graft (or not if performed by distraction osteogenesis) or requires removal of a wedge of bone (closing wedge). If an osteotomy is carried out in the plane of deformity, it allows correction of the deformity by rotating the opposing bone surfaces while maintaining maximum bone contact. Where the deformity is both angulation and rotation, the true plane of deformity is oblique to both the long axis and the transverse plane. Osteotomy in this plane allows rotation of opposing flat surfaces that correct angular and rotational deformity simultaneously and maintain full bone contact. We present a simple graphical method for determining this total plane of deformity and the plane of osteotomy about which the total deformity can be corrected.

## Method

The following is the description of the full planning method.

### Measurement of deformity

**Fig. 1:**
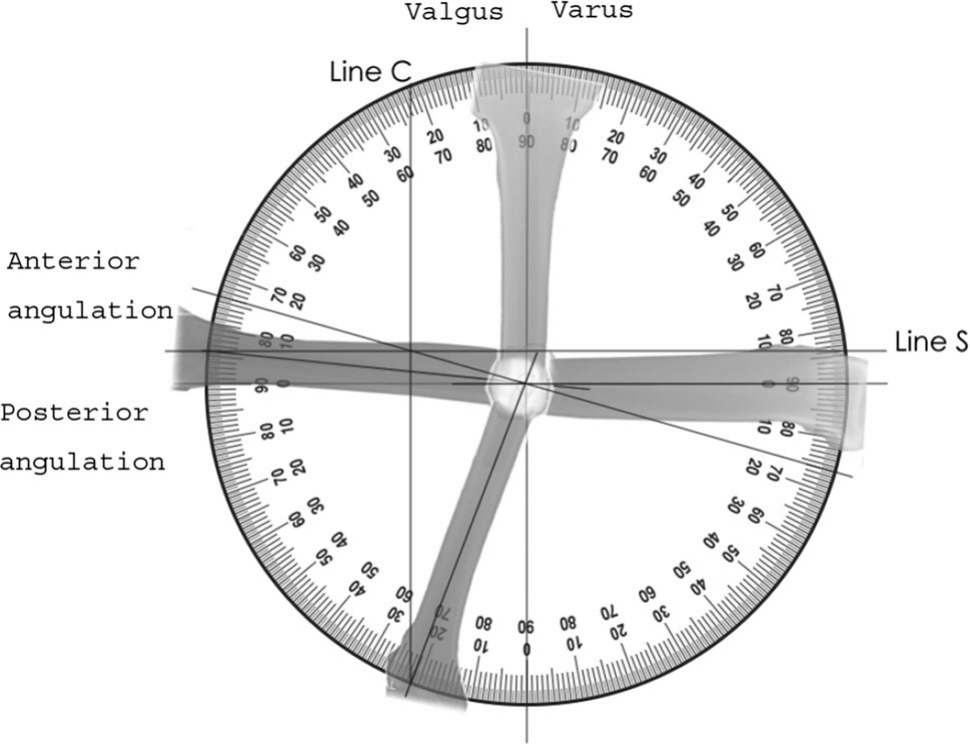
Angular deformity in anterior–posterior and lateral planes

The angular deformity is measured in degrees on the AP radiograph (coronal angle C) and lateral radiograph (Sagittal angle S) using standard methods (Fig. 1). A CT scan of both the affected and normal limb is used to measure the rotational deformity (rotational angle R). The rotational angle can also be measured using clinical measurement. This method combines deformity measured on coronal, sagittal and axial images and resolves them into a single oblique plane. The coronal and sagittal planes are first resolved into the true angular deformity in the oblique plane (of the longitudinal axis), and this is combined with axial angulation (the rotational deformity, which is in effect an angular deformity in the axial plane) to determine the total deformity in a different plane.

Part one calculates the plane and magnitude of angular deformity from the AP and lateral radiographs giving the total deformity in the longitudinal plane. Part two calculates the total deformity by combining rotational and angular deformity.

**Fig. 2:**
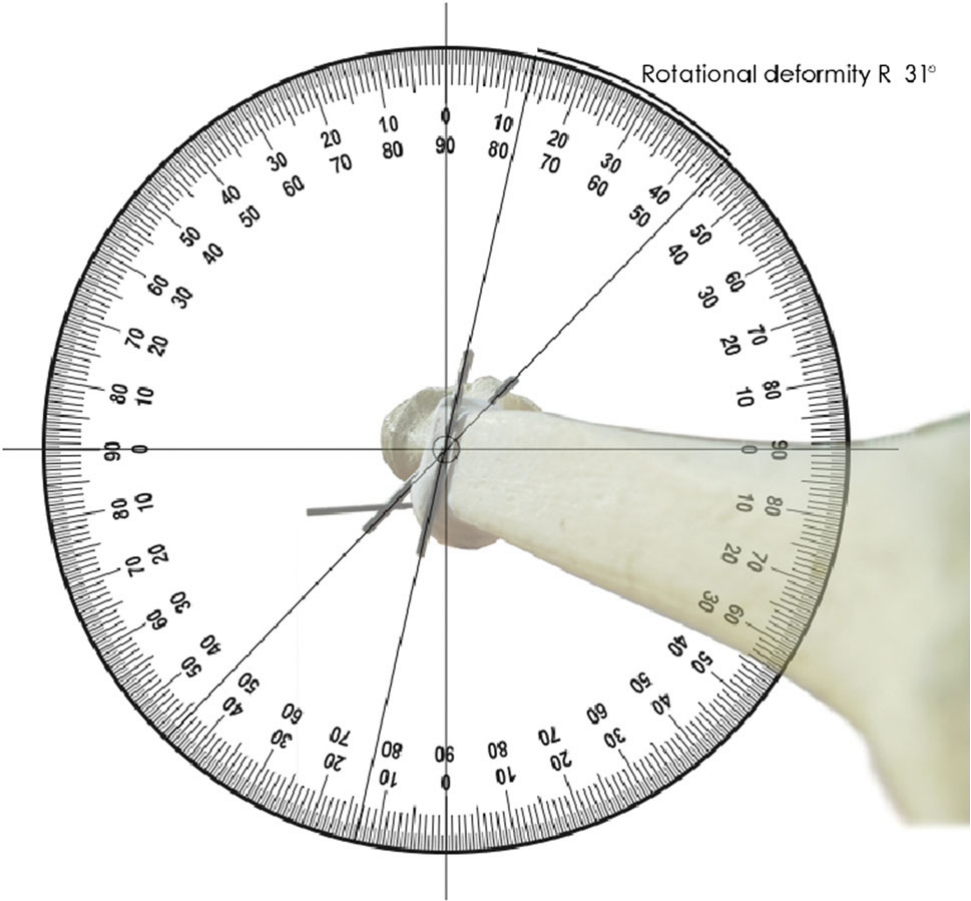
Rotational deformity in axial plane

The method is described using a worked example; the AP radiograph (Fig. 1) shows a valgus angulation (C) of 21° for a right tibia, the lateral radiograph shows an anterior angulation (S) of 6° and the axial imaging shows a 31° external rotation deformity (Fig. 2 which shows rotation markers when looking down onto a right tibia).

### Part one

The simplest method of measuring the true magnitude of angular deformity is to image the deformity across the plane of maximal angulation and measure it directly. Alternatively, the magnitude and plane of deformity can be calculated from measurements taken from true AP and lateral radiographs. This can be done graphically:

The coronal plane deformity is drawn as a vertical line (Line C) Fig. 1. This vertical line intersects the circumference of the protractor at the measured angle C (21°) subtended from the central point starting at the AP axis (0°). For a small angular deformity, line C lies close to the AP axis. For a bigger angular deformity, line C is deflected further from the AP axis. For a valgus deformity, Line C is drawn towards the lateral side and varus is drawn on the medial side.

The process is repeated for the sagittal plane deformity. The sagittal plane deformity is drawn as a horizontal line (Line S) Fig. 1. This horizontal line intersects the circumference of the protractor at the measured angle S (6°) subtended from the central point starting at the horizontal axis (0°). For a small angular deformity, line S lies close to the horizontal axis. For a bigger angular deformity, line S is deflected further from the horizontal axis. For a recurvatum (anterior angulation), deformity line S is drawn towards the anterior side and if procurvatum (posterior angulation) it is drawn on the posterior side.

**Fig. 3:**
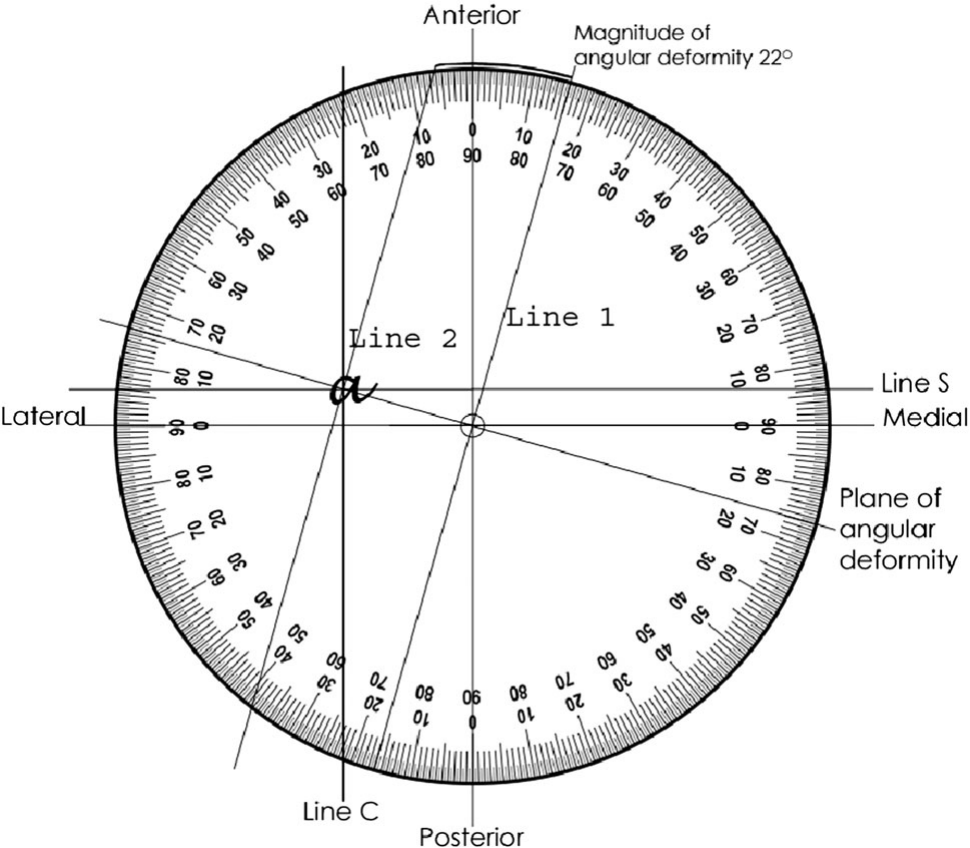
Plane and magnitude of angular deformity

A line is drawn from the central point through the intersection of Line C and Line S. This represents the resolved plane of angular deformity. Line C and Line S intersect at point *a* (Fig. 3). In this example the plane of angular deformity is 16° to the coronal plane.

To measure the magnitude of deformity, two lines are drawn at right angles to the plane of angular deformity (Fig. 3). Line 1 is drawn through the central point and Line 2 is drawn through the intersection. The magnitude of angular deformity is the separation of these lines measured in degrees at the circumference.

The plane of angulation is 16° anterior to the coronal plane or 74° external to the sagittal plane. The magnitude of angular deformity is measured as 22° (A). An AP radiograph taken with the leg 16° externally rotated would demonstrate the full angular deformity (A) of 22°.

### Part two

The angular and rotational deformities are now resolved into a final single plane. The starting position for this is the bisector of the angulation in the plane of the first resolved angular deformity.

Step one is to define the bisector of the angular deformity. This is calculated [90 + (A/2)] for the convexity and [90 − (A/2)] for the concavity of the deformity. In this example it is 101° to both proximal and distal segments in the plane of the angular deformity when viewed from the apex of the convexity. The bisector is marked by a K-wire in the plane of angular deformity. *A simple line diagram will help understand this part.*

**Fig. 4:**
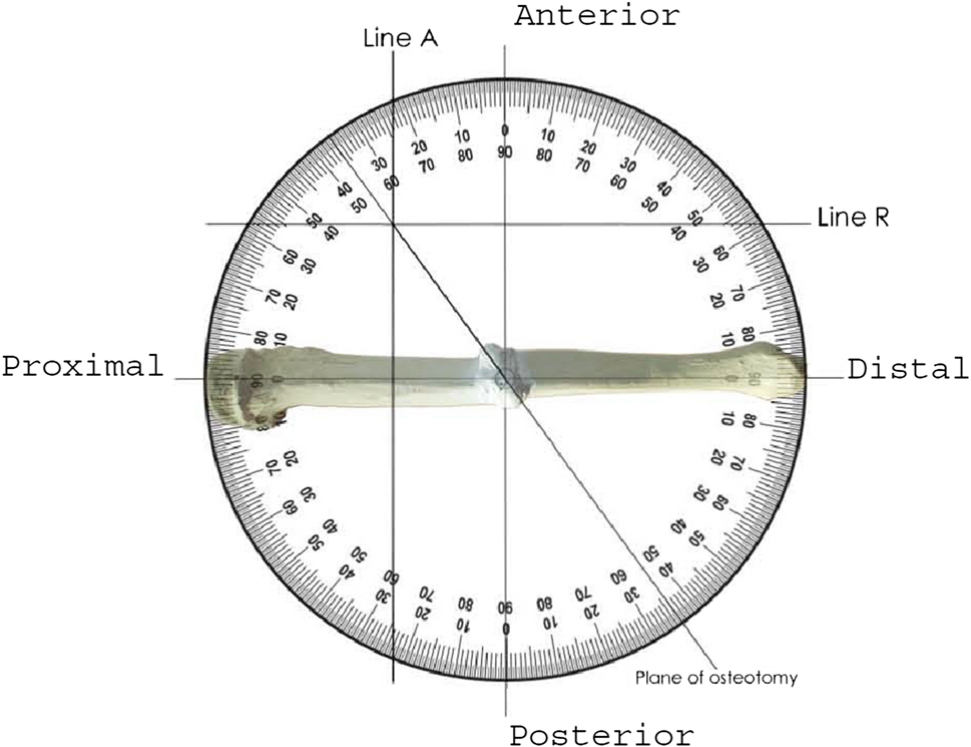
Plane of osteotomy

A photocopy of a standard circular protractor is marked proximal, distal, anterior and posterior with axes intersecting at the central point (Fig. 4). The horizontal axis is marked proximal and distal matching the operative view of the bone. In this example proximal is marked on the left and distal on the right.

The horizontal axis on the protractor lies in the plane of angular deformity; this means that when viewing the bone as in the example on Fig. 4, the bone has been rotated such that the true plane of angular deformity faces the viewer. In this example, the bone has been rotated 16° externally. The AP axis now lies at right angles to the bisector of the angular deformity. The central point is looking down the axis of the bisector of angular deformity.

The resolved angular deformity of 22° in the oblique plane (A) is represented by (line A) in Fig. 4. A vertical line is drawn that intersects the circumference of the protractor at the measured angle (22°) from the central point starting at the AP axis. For a deformity approached from the convexity, as in this example, this is marked to the proximal side but if seen from the concavity it is marked to the distal. In this example line A is drawn proximal from the AP axis.

The process is repeated for the rotational deformity which is drawn as a horizontal line (line R) in Fig. 4. This horizontal line intersects the circumference of the protractor at the measured angle R (31°) subtended from the central point starting at the horizontal axis (0°). For a small rotational deformity, line R lies close to the horizontal axis. For a bigger angular deformity, line R is deflected further from the horizontal axis. External rotation is drawn anterior to the horizontal axis and internal rotation is drawn posterior to the horizontal axis. In this example line R is anterior.

A straight line is drawn from the central point to the intersection of line A and line R and continued to the circumference. This defines the plane of osteotomy. In this example it is measured as 54° from the horizontal axis or plane of angular deformity and 36° from the transverse plane rotated about the bisector of angulation.

For a deformity that is mainly angular, the final plane is close to the plane of angular deformity (horizontal axis). For deformity that is mainly rotational, the plane is closer to the transverse or axial plane (vertical axis).

If this convention is not followed correctly, it is possible to plan an osteotomy that achieves a mirror image of the desired correction, creating, for example, external rotation with varus rather than internal rotation with varus. The osteotomy rule that confirms the correct orientation is: the apex (acute angle) of the distal fragment rotates towards the concavity of the angulation. If this rotation corrects the rotational deformity, the osteotomy is correctly orientated.

### Surgical technique

The starting point for the osteotomy is the bisector of the angular deformity in the plane of the angular deformity (the resolved oblique plane performed in part one). The deformity is imaged in the plane of maximum deformity by rotating the limb until the maximum angular deformity is seen. A Kirschner wire (K-wire) is then placed along the bisector in the plane of angular deformity.

The saw blade is rotated from longitudinal to transverse around this bisector axis depending on the combination of rotation and angulation. The more rotation the more transverse the cut, the more angulation the more longitudinal the cut. The calculation of the combination of rotation and angulation to provide the final plane uses the circular protractor method (part two).

The osteotomy rule to correctly orient the cut is: the apex (acute angle) of the distal fragment rotates towards the concavity of the angulation. This osteotomy rule is critical as it is easy to rotate the saw blade to produce a mirror image of the desired cut that increases the rotation deformity with correction of angulation.

**Fig. 5:**
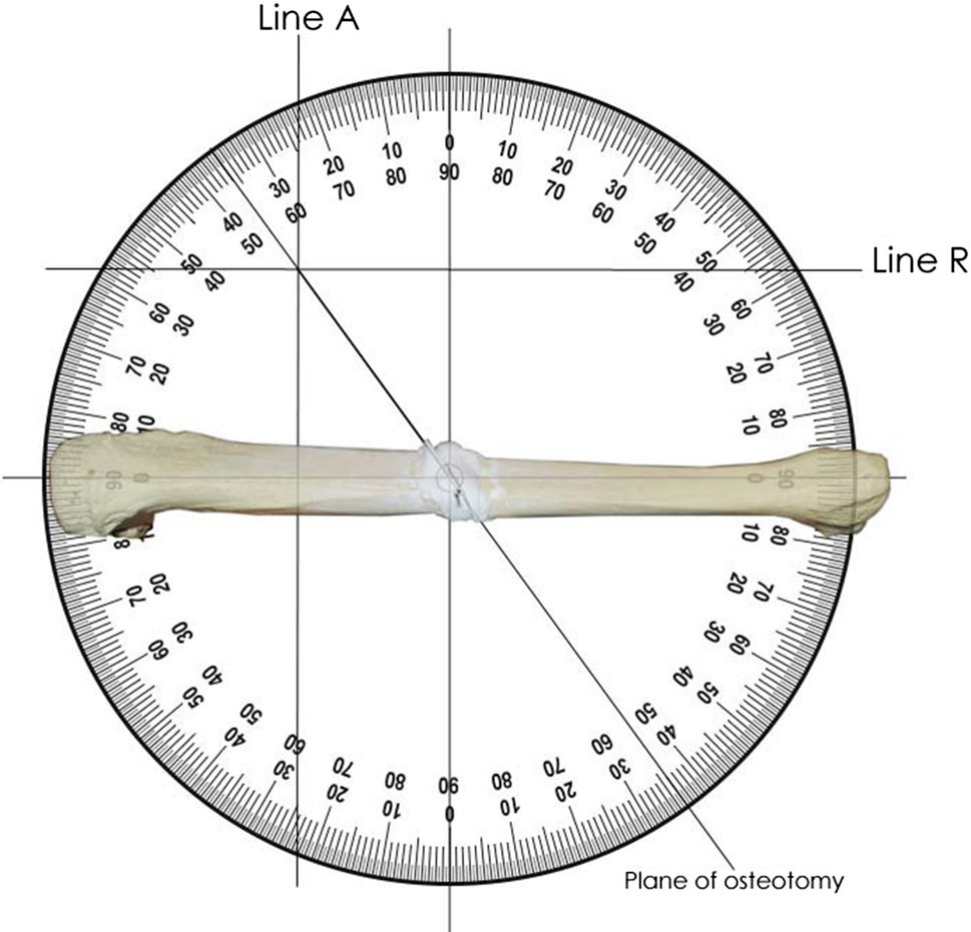
Full correction of angular and rotational deformity following rotation around the osteotomy plane

Figure 5 shows the full correction of angular and rotational deformity following rotation around the osteotomy plane.

## Discussion

Oblique plane osteotomy has been described for the correction of Blount’s disease, proximal femoral deformity, metatarsal deformity and the correction of tibial malunion [1, 2].

A single-plane osteotomy has a number of advantages over a wedge osteotomy: the second cut of a wedge osteotomy is difficult to make accurately once the bone is divided, making it easy to create a new deformity; it is possible to under-correct the original deformity; and an incorrectly created wedge can produce a bone gap. Resection of a bone wedge creates shortening that can be avoided by the use of a single-plane osteotomy. Obliquity of this single-plane osteotomy often allows stable inter-fragmentary screw fixation with compression and a large area of bone contact that is not possible with a transverse osteotomy. Translation along the plane of osteotomy allows correction of translational deformity with restoration of length. Where the surgical anatomy dictates the need for an approach to the concavity of angulation, a closing wedge may be technically challenging, whereas a single cut and rotation about a single oblique plane provides an elegant solution.

When a hexapod external fixator is used to simultaneously correct angular and rotational deformity, the segments are rotated about this single plane. If an osteotomy can be performed in this plane, the cut bone surfaces rotate about each other without creating a bone gap [3].

The starting points for describing this plane of osteotomy are the plane of angular deformity and the bisector of the angular deformity. These are both relatively easy to define and identify using the image intensifier in theatre. Rotation of the saw blade is made around the axis that is the bisector of the angular deformity. This method of planning can also be used to identify the correct plane for a focal dome osteotomy, the axis of which lies at right angles to the final osteotomy plane described. A deformity that is predominantly angular with little rotation exists predominantly in the long axis of the bone, and therefore, a focal dome osteotomy may be preferred.

When rotating about the final plane, assuming the proximal segment remains static, the movement of the distal segment is through the arc of a cone, the central axis of which is at 90° to the plane of osteotomy. The correction of angulation therefore occurs in a circular movement and not in a straight line. The use of the bisector of angulation as the initial reference corrects for this circular movement to produce the desired correction. If the chosen plane is parallel to the bisector but not accurately rotated, the angular correction is achieved with either excessive or insufficient rotation. If the chosen plane is not parallel to the bisector, rotational correction is accompanied by angulation in a different plane.

Other methods of describing this plane of osteotomy reference from the plane of the proximal or distal segment as the starting point and require two re-orientations of the blade to find the plane of osteotomy with the second reorientation towards the direction of rotation by half the rotational deformity. Any inaccuracy with this reorientation may cause significant angular deformity.

The graphical method of combining angulation on AP and lateral radiographs using a circular protractor has been shown to produce results to within 1.5° of the correct mathematical results. This graphical method of calculating the plane of osteotomy is accurate for deformities up to 90°, whereas the vector method of planning is less accurate when the total deformity exceeds 30°. This method may be applied to deformity correction in any bone where rotational and angular deformities are combined and considerably simplifies the planning of single-plane osteotomy for the correction of complex deformity.
